# Ancient Microbiomes as Mirrored by DNA Extracted From Century‐Old Herbarium Plants and Associated Soil

**DOI:** 10.1111/1755-0998.14122

**Published:** 2025-05-24

**Authors:** Gianluca Grasso, Régis Debruyne, Martino Adamo, Olivier Rué, Franck Lejzerowicz, Lucie Bittner, Valeria Bianciotto, Roland Marmeisse

**Affiliations:** ^1^ Department of Life Science and Systems Biology (DBIOS) Università Degli Studi di Torino Torino Italy; ^2^ Institut de Systématique, Evolution, Biodiversité (ISYEB), Muséum National D'histoire Naturelle, CNRS Sorbonne Université, EPHE, Université Des Antilles Paris France; ^3^ Institute for Sustainable Plant Protection (IPSP) National Research Council (CNR) Torino Italy; ^4^ Bioarchéologie, Interactions Sociétés Environnements (BioArch: UMR 7209 CNRS‐MNHN) Muséum National d'Histoire Naturelle Paris France; ^5^ MaIAGE Université Paris‐Saclay, INRAE Jouy‐en‐Josas France; ^6^ BioinfOmics, MIGALE Bioinformatics Facility Université Paris‐Saclay, INRAE Jouy‐en‐Josas France; ^7^ Section for Aquatic Biology and Toxicology University of Oslo Oslo Norway; ^8^ Institut Universitaire de France Paris France

**Keywords:** ancient DNA, herbaria, paleomicrobiology, plant microbiomes

## Abstract

Numerous specimens stored in natural history collections have been involuntarily preserved together with their associated microbiomes. We propose exploiting century‐old soils occasionally found on the roots of herbarium plants to assess the diversity of ancient soil microbial communities originally associated with these plants. We extracted total DNA and sequenced libraries produced from rhizospheric soils and roots of four plants preserved in herbaria for more than 120 years in order to characterise the preservation and taxonomic diversity that can be recovered in such contexts. Extracted DNA displayed typical features of ancient DNA, with cytosine deamination at the ends of fragments predominantly shorter than 50 bp. When compared to extant microbiomes, herbarium microbial communities clustered with soil communities and were distinct from communities from other environments. Herbarium communities also displayed biodiversity features and assembly rules typical of soil and plant‐associated ones. Soil communities were richer than root‐associated ones with which they shared most taxa. Regarding community turnover, we detected collection site, soil versus root and plant species effects. Eukaryotic taxa that displayed a higher abundance in roots were mostly plant pathogens that were not identified among soil‐enriched ones. Conservation of these biodiversity features and assembly rules in herbarium‐associated microbial communities indicates that herbarium‐extracted DNA might reflect the composition of the original plant‐associated microbial communities and that preservation in herbaria seemingly did not dramatically alter these characteristics. Using this approach, it should be possible to investigate historical soils and herbarium plant roots to explore the diversity and temporal dynamics of soil microbial communities.

## 1 Introduction

The contribution of the soil microbiome to soil and plant health is widely recognised (Banerjee and van der Heijden 2022; Bardgett and van der Putten 2014). A current challenge is to evaluate how ongoing global changes are affecting the diversity of these microbial communities, with potential consequences on their contribution to ecosystem processes. This challenge is essentially addressed in short‐term studies that compare microbial communities along environmental gradients or in manipulated mesocosms or field experiments. From these studies emerge a number of common signatures (e.g., decrease in host specificity, increase in abundance of rare microbes, pathogens and hypermutators), but also differences in the response of soil microbiomes to environmental constraints (e.g., shifts in pH, temperature and humidity values; Berg and Cernava 2022; Zhou et al. 2020). These short‐term studies, which rely on high‐throughput sequencing techniques to evaluate the microbial diversity, have almost all been carried out in the last 15 years in the 21st century (Zhou et al. 2020). These studies therefore monitor short‐term responses to environmental changes of soil microbial communities that have already experienced several decades of global changes. This is particularly true in the case of arable soils, which in different parts of the world have been managed since the late 1940s–1960s by intensive farming practices. These practices broke completely with the former ones, which did not depend on the use of chemical fertilisers and pesticides to grow high‐yielding crop varieties.

In this context, we can ask what the long‐term consequences on soil microbiomes of intensive farming practices have already been. Indeed, several ecological processes occur at a pace incompatible with short‐term monitoring. This is the case of biological invasions and diversity impoverishment, two facets of the global biodiversity crisis. This crisis has been put forth and quantified by studies targeting animals and plants (Nic Lughadha et al. 2020; Pyšek et al. 2020; Rull 2021). In the case of microorganisms, invasions—but rarely extinctions—are documented essentially for symptom‐producing plant or animal pathogens, for a few mushroom‐producing fungal soil saprotrophs as well as for bacterial or fungal root symbionts (Desprez‐Loustau et al. 2007; Thakur et al. 2019; Wang et al. 2023). No data are available for the great majority of ‘elusive’ soil microorganisms. Agricultural practices such as the release of microbial inoculants (Liu et al. 2022) or mass movements of entire microbiomes through long‐distance trade and travel (Zhu et al. 2017) suggest, however, that widespread invasion of soil ecosystems by alien microbial species and genetic elements is likely.

In order to evaluate long‐term changes in the composition of soil microbial communities, it is necessary to access ancient soil samples and to implement appropriate protocols to reveal the composition of their original microbiomes. Regarding the later prerequisite, molecular paleomicrobiology, based on the analysis of degraded ancient DNA (aDNA) extracted from historical samples, is certainly the most relevant approach (Grasso, Bianciotto, et al. 2024; Schubert et al. 2024). Paleomicrobiology allows for a diachronic approach to the evolution of microbial diversity (Arriola et al. 2020). It allows documenting taxonomic and functional diversity of microorganisms in ancient, up to tens of thousands year‐old items preserved in favourable environments such as caves or permafrost (Waldrop et al. 2023). It has been implemented for samples as diverse as archaeological artefacts such as dental calculus and desiccated or mineralised faeces (Hagan et al. 2020), thus giving access to ancient human/animal oral and intestinal microbiomes (Brealey et al. 2021; Fellows Yates et al. 2021; Maixner et al. 2021; Quagliariello et al. 2022). These data have been used to infer the diet and local environmental conditions to which were exposed the corresponding hosts. Regarding ‘non‐host’–associated environmental samples, the analysis of aDNA preserved in stratified marine or freshwater sediments recapitulates the temporal dynamics of aquatic microbial communities and how they responded to past environmental changes (Barouillet et al. 2022; Keck et al. 2020; Siano et al. 2021).

Regarding archived soil samples, although collections do exist, the corresponding samples have, for most of them, been excavated in the last 50 years. Older referenced samples are rare (e.g., the Vasily Dokuchaev Museum of Soil Science, St. Petersburg, Russia) and sometimes limited to very few collection sites, as exemplified by the Rothamsted long‐term agronomic experiments that started in the 1840s and for which soil samples were continuously collected (Poulton 2011). We recently identified herbaria as potential repositories of (rhizospheric) soils that were left attached to the roots of many herbaceous plants preserved in these collections (Bianciotto et al. 2022). These collections that may encompass more than 400 million plant samples globally (https://sweetgum.nybg.org/science/wp‐content/uploads/2023/11/The_Worlds_Herbaria_2022.pdf) have been mostly established in the 19th and first half of the 20th century, a period that predates the so‐called ‘great acceleration’ in global changes (Steffen et al. 2015). For many plant species, herbaria can contain hundreds of different samples collected over the entire species' distribution areas and several decades. These collections can thus be used to trace plant adaptation to global changes through time, from anatomical, physiological and genomic points of view (DeLeo et al. 2020; Denney and Anderson 2020; Lang et al. 2022; Burbano and Gutaker 2023).

Initial studies have targeted plants in herbaria to study their original microbiomes using metabarcoding (Daru et al. 2018; Heberling and Burke 2019) or shotgun sequencing (Bieker et al. 2020; Weiß et al. 2016) of plant‐extracted DNA. We identified two studies that investigated the microbiomes of archived dried soil samples (Clark Ian and Hirsch 2008; Ivanova et al. 2017). Both of these studies relied on metabarcoding, an approach now criticised in paleomicrobiology as the size of amplified DNA sequences usually far exceeds the average size of extracted historical DNA (Orlando et al. 2021), thus potentially favouring amplification of DNA from external contaminants or from taxa producing resistant resting cells. The objective of the present study is to extend the approach of shotgun‐sequencing libraries built from total DNA to herbarium plant‐associated desiccated rhizospheric soils, in order to access the past diversity of the soil microbiome and ultimately infer its time‐dependent response to environmental changes. This experimental framework needs first to be validated from both experimental and analytical points of view. It is necessary to evaluate the authentic nature of the extracted DNA and to demonstrate that the deduced biological data are in line in terms of biodiversity and community assembly rules with the data commonly reported for extant soil microbiomes. For this purpose, we selected four cultivated and taxonomically diverse plants: *Lactuca sativa* (lettuce) and three cereals, oat, rye and durum wheat. To minimise geographic variability, which has a major impact on soil microbial community diversity and may blur other drivers of community assembly, we focused on a rare herbarium plant collection where plants were grown in a common garden in the suburbs of Paris (France) and collected in the first years of the 20th century, that is about 120 years before the start of the study.

## 2 Materials and Methods

### 2.1 Herbarium Specimens

**FIGURE 1:**
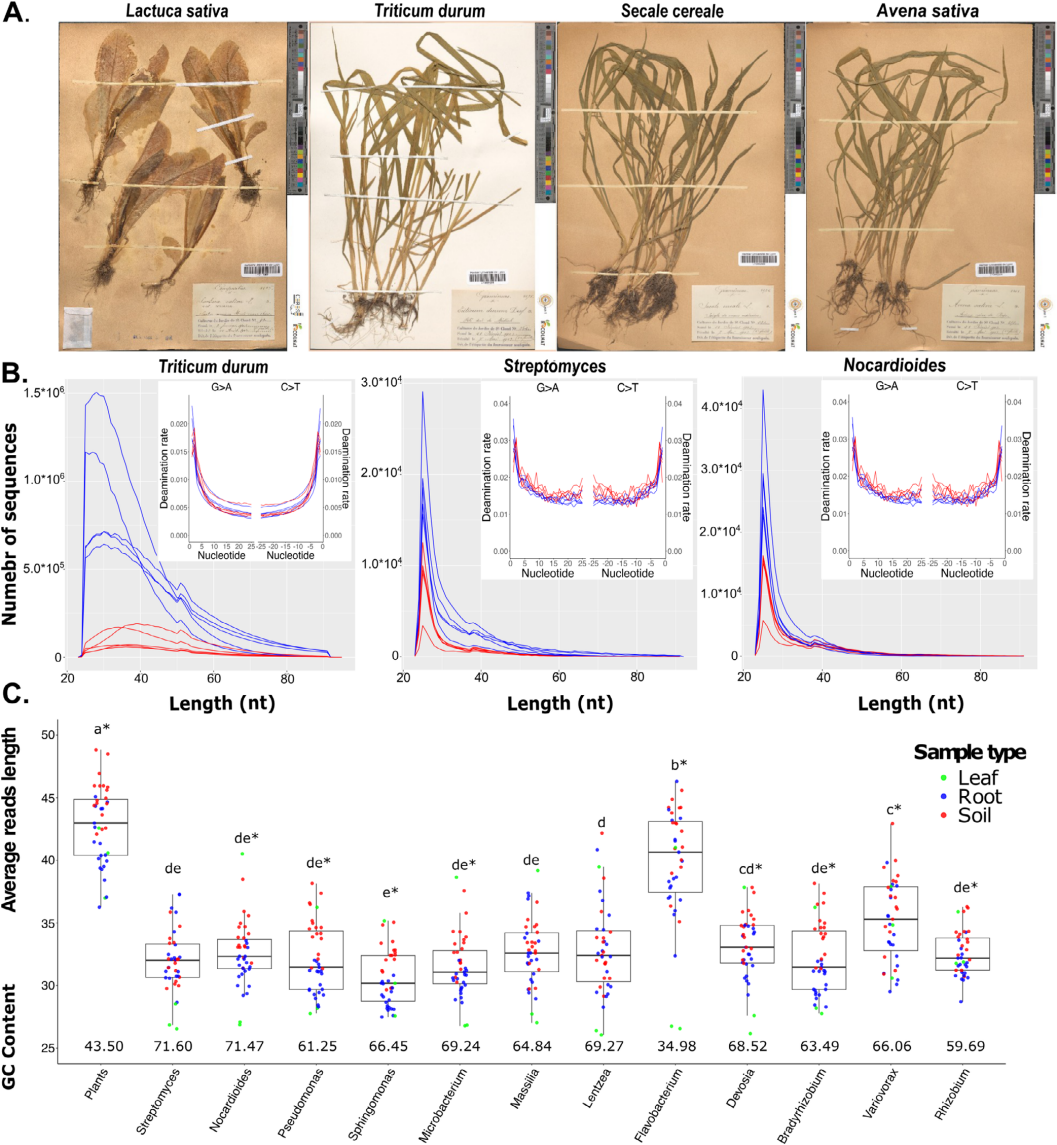
Analysis of DNA extracted from herbarium samples highlights typical signatures of ancient DNA and suggests plant/soil and species‐dependent effects on patterns of degradation. (A) An illustration of the herbarium plants with associated soil used for the experiments; numbers in parenthesis refer to their ‘RecolNat’ accession numbers (https://explore.recolnat.org/search/botanique/type=index). (B) Length distribution and deamination profiles of DNA fragments (merged reads) extracted from *Triticum durum* roots (blue curves) and associated soil (in red) affiliated to *T. durum* , *Streptomyces* and *Nocardioides*. Deamination profiles are illustrated for the first 25 nucleotides at the 5′ end (G to A transitions) and 3′ one (C to T transitions). (C) Distribution (box plots) of the average lengths of the sequenced DNA fragments extracted from 39 leaf (green), root (blue) and soil‐associated (red) herbarium specimens. Each box plot refers to the fragments assigned to either a plant genome (4 species) or one of the 12 most abundant bacterial genera. Different letters below the plots indicate statistically supported (*p* < 0.001) differences between taxa, while asterisks indicate, for each of the taxa, significant (*p* < 0.05) differences in the average length of the fragments extracted from soil and plant tissues.

All plant specimens (Table S1, Figure 1A), four or five per plant species, came from the Bonaparte's collection detained by the herbarium of the University of Lyon (herbarium code, LY), France and can be visualised on the RECOLNAT website (https://explore.recolnat.org). Specimens belonged to the species *Avena sativa* , *L. sativa* , *Triticum durum* and *Secale cereale* . Most plants (16 out of 19) were originally cultivated in the same garden, in ‘Saint‐Cloud’ in the suburbs of Paris, a site that has now been urbanised. The remaining three specimens were collected in three other sites in France (Table S1). Several plants grown in Saint‐Cloud are thought to be genetically distinct, as they belonged to a specific variety mentioned on the herbarium's labels. Plants were collected over a short period of time, between the years 1903 and 1907.

For each specimen, about 20 fine root fragments—ca. 0.5 cm long—were collected using sterile tweezers and scalpel blades. Soil particles aggregated around the roots (between 10 and 100 mg per specimen) were detached from the roots and collected separately. For two of the *L. sativa* plants, no rhizospheric soil was present around the roots. For three specimens (belonging to *L. sativa, S. cereale* and *T. durum* ), leaf fragments were also sampled. The collected material was transferred into sterile tubes and stored at room temperature until DNA extraction.

### 2.2 DNA Extraction Manipulation and Sequencing

DNA extraction and its manipulation were performed in the dedicated aDNA facility at the ‘Musée de l'Homme’ in Paris (https://www.ecoanthropologie.fr/fr/paleogenomique‐et‐genetique‐moleculaire‐6206). The platform is equipped with clean rooms under positive air pressure and UV‐sterilised daily to prevent contamination from the outside environment. Strict separation is maintained between spaces dedicated to sample preparation, DNA extraction, control, amplification and sequencing library preparation. Laboratory coveralls were worn for all experiments and specific cleaning procedures of equipment and spaces were implemented (2.6% bleach and/or 20 min UV (256 nm) cross‐linking).

Total DNA was extracted following the method of Murchie et al. (2021) optimised for ancient sediment samples, with minor modifications (Grasso, Rotunno, et al. 2024). Three control extraction and library preparation blank samples were systematically performed to mitigate the risk of in‐laboratory contamination. End‐repaired DNA extracts were used to prepare Illumina libraries using a double‐indexing PCR with a variable number of 9–14 cycles (Kircher et al. 2012) per individual library (Table S4). All library blanks were submitted to 14 cycles of amplification but failed to produce any detectable product, except for low proportions of adapter dimers. Amplified libraries were characterised by fluorometric quantification (Qubit II, Invitrogen) and capillary electrophoresis (Labchip GX Touch, Perkin‐Elmer) and pooled in equimolar amounts. The multiplexed libraries were sequenced on an Illumina NovaSeq 6000 instrument (SP flowcell, 800 × 10^6^ paired‐end 2 × 50 bp reads) at the iGenSeq sequencing facility (https://igenseq.institutducerveau‐icm.org/).

### 2.3 Sequence Filtering, Quality Control and Read Mapping

Reads were subjected to quality control, adapter trimming and merging using FastQC v.0.12.0 (Andrews 2010) and fastp v.0.23.1 (Chen et al. 2018). For the trimming, default commands were used except for two options to account for the short length of the reads: *‐‐length_required* option was set to 25 nucleotides and *–overlap_len_require* was lowered from 30 to 10 nucleotides. Reads were then mapped using BWA v.0.7.17 (Li and Durbin 2009) to the corresponding plant reference genomes (Table S2) to filter out the plant sequences. The pool of unmapped reads that includes the host plant microbiome (e.g., eubacteria, archaea, fungi, micro‐eukaryotes, viruses) was extracted using Samtools v.1.14 (Danecek et al. 2021). The unmapped reads were further mapped to the hg28 human reference genome (accession no. GCF_000001405.26) to exclude any human contamination.

### 2.4 aDNA Validation and Comparison to Modern Microbiomes

The rate of cytosine deamination at the extremities of the reads was assessed using MapDamage2 (Jónsson et al. 2013) based on the frequencies of transitions and transversions in alignments to reference genomes. MapDamage was separately run on the plant reads and on the reads affiliated to the 12 most abundant bacterial genera identified in the microbiome dataset using reference genomes from NCBI (April 2023; Tables S2 and S3). Since reference bacterial genomes are related but may not be identical to those of the taxa present in the samples, this results in a high background noise in the mapping output that can ultimately mask base substitutions at the 3' and 5' ends of the DNA molecules. For this reason, MapDamage was run only for bacterial genera with more than 3.10^5^ reads in the global dataset (i.e., on average 8000 reads per sample). For both plants and bacteria, the distribution of metagenomic fragment lengths inferred from the pool of merged R1 and R2 for each library was compared for the different groups using ANOVA testing in R 4.2.2 (R Core Team 2021).

Microbiome reads were compared with modern metagenomes from the Earth Microbiome Project (EMP, Thompson et al. 2017) using the Qiita platform (Gonzalez et al. 2018). Qiita offers access to a supercomputer to upload, search and analyse metagenomic samples jointly using Qiime2 (v.2023.2, Bolyen et al. 2019). Herbarium metagenomes were uploaded to Qiita, merged with the EMP samples, aligned using Bowtie2 (Langmead et al. 2009) to the WoLr2 database (‘Web of Life’ release 2) and classified using Woltka (v.0.1.4, Zhu et al. 2022) to obtain feature tables across the same WoL reference genomes. These tables were used to measure alpha and beta diversity into Qiita, using Qiime2. To explore differences in composition and diversity among various microbiomes, the core_metrics function of Qiime2 v.2023.2.0 (Bolyen et al. 2019) was employed.

### 2.5 Analysis of Microbial Community Composition and Diversity

The microbiome reads were taxonomically annotated using Kaiju v.1.9.0 (Menzel et al. 2016), which confronts translated sequences to a database of bacterial, archeal and protist (including fungi) proteomes (nr_euk data bank release 2021–03, https://github.com/bioinformatics‐centre/kaiju). Abundance tables at phylum and genus levels were obtained and further explored in R. The Shannon alpha diversity metric was calculated for each sample using the fossil package v.0.4.0 (Vavrek 2011) and the vegan package v.2.6–4 (Oksanen et al. 2022). Observed differences were evaluated using the Kruskal–Wallis test. For beta diversity, a Bray–Curtis dissimilarity matrix was calculated using *vegan* based on feature tables created using *fossil* and Hellinger transformed using *labdsv* 2.1‐0 (Roberts 2023). This matrix was explored with Principal Coordinates Analysis (PCoA, Anderson and Willis 2003) using *ape* v.5.7‐1 (Paradis et al. 2004). Permutational analyses of variance (PERMANOVA) were conducted using the ‘adonis’ function of *vegan*, scrutinising community structure across taxa (genus and phylum levels), collection sites and sample type (soil vs. root). Diversity measures for Bacteria–Archaea and Eukarya were repeated both at phylum and genus levels. Several statistical approaches—namely DESeq2 v.1.26.0 (Love et al. 2014), ANCOM‐bc2 (Lin and Peddada 2020) and ALDEx2 (Fernandes et al. 2013)—were implemented to detect differentially abundant genera across sample types.

## 3 Results

### 3.1 DNA Extraction, Sequencing and Sequence Analysis

DNA was successfully extracted from all 39 herbarium leaf, root and soil samples representing four plant species collected in four geographic sites in France *ca* 120 years ago. Total amounts of extracted DNA ranged from 64 to 398 ng for root/leaf samples and from 787 to 1203 ng for soil samples. Electrophoretic separation of amplified library DNA fragments produced, in all cases, a peak lower than 200 bp, corresponding to a majority of DNA inserts shorter than 70 bp, thus justifying a NovaSeq 6000 run of only 50 cycles in paired end. After demultiplexing, quality control, paired reads merging and elimination of sequences shorter than 25 bp, we obtained an average of 21.8 ± 7.7 (min–max: 8.5–40.3) millions of merged reads per sample (Figure S1A). On average, 67.5% (50.5–85.4) of the merged fragments were shorter than 50 bp. The mean DNA fragment length for the different sample types was 40.7, 42.1 and 48.0 bp for the leaf, root and rhizosphere samples, respectively (Figure S1B).

The average read duplication rate was only on average 3% per library. The average percentage of reads mapping onto plant genomes varied between 87.5% (86.8–88.6), 62% (24.09–85.9) and 23.54% (3.8–49.8) for leaves, roots and soil samples, respectively (Table S4). On average, 0.4% of reads mapped to the human genome with only three samples that had percentages above 0.7% (Table S4).

Size distributions were evaluated separately for the sequences that mapped onto the host plant genome and for the microbiome reads that mapped onto genomes of the 12 most represented bacterial genera (Figures S2 and S3). These 12 genera belong to three different phyla (Proteobacteria, Actinobacteria and Bacteroidetes). Sample average length of merged reads (i.e., the size of extracted DNA fragments) never exceeded 50 bp, and statistically supported (*p* < 0.001, ANOVA) differences between sample types (Figure 1C) were observed. On average, plant DNA fragments were ca. 10 nucleotides longer than microbiome DNA fragments. The length of the microbiome fragments also differed between bacterial genera. Fragments attributed to *Flavobacterium* were ca. 5–15 nucleotides longer than fragments attributed to the other bacterial genera. Of the twelve microbial taxa, *Flavobacterium* had the lowest genome GC content (around 35% vs. 59%–72% for the other 11 bacterial genera, Figure 1C). The sample type (root, rhizospheric soil or leaf) from which DNA was extracted had a significant effect on DNA fragment size. Except for sequences that mapped to *Streptomyces*, *Massilia* and *Lentzea*, DNA extracted from soil was less fragmented than plant DNA or than microbial DNA extracted from plant tissues (roots or leaves, *p* < 0.05, ANOVA Figure 1B).

Besides high fragmentation, another typical feature of aDNA is cytosine deamination. This damage preferentially occurs at DNA fragment overhangs and results in higher apparent C‐to‐T and G‐to‐A substitutions at the 5' and 3' ends of the molecules, respectively. Substitutions were quantified for the fragments mapping to four host plant genomes or to the 12 most abundant bacterial genera (Figures S4 and S5). In all samples and for all taxa, the rates of C‐to‐T and G‐to‐A substitutions were higher than background at the 5' and 3' terminal positions, respectively, and displayed typical curves for aDNA libraries when progressing inward (illustrated in Figure 1 for *T. durum* and two representative bacterial genera). For plant sequences, substitution rates at the first position of the DNA fragments were in the range of 0.014–0.022, while they were slightly higher (0.022–0.038) in the case of bacterial sequences.

### 3.2 Below Ground Herbaria Microbial Communities Cluster With Extant Soil Ones

**FIGURE 2:**
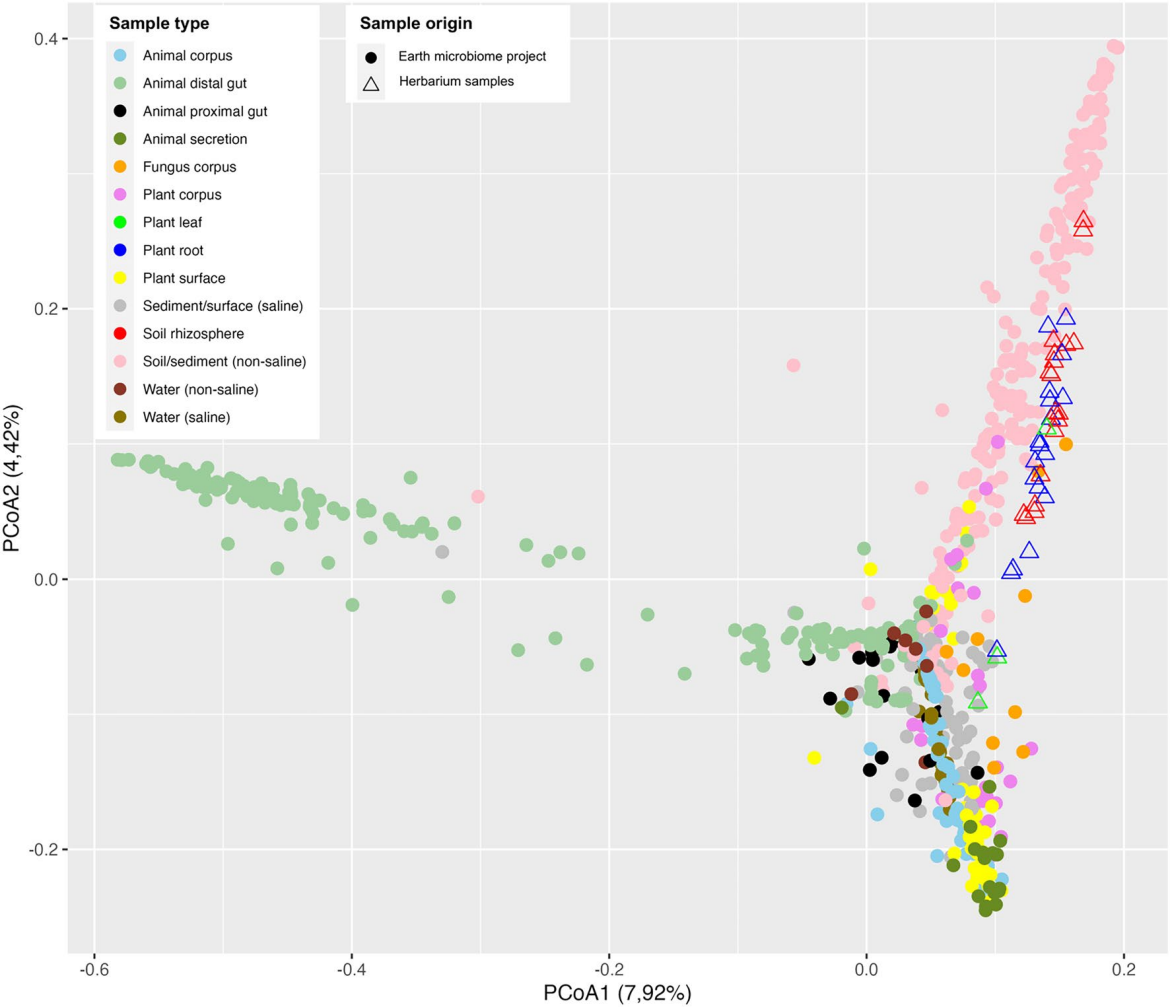
Herbarium‐associated soil and root microbial communities cluster with extant soil communities. Herbarium soil and root microbial communities (empty triangles) cluster close to extant soil communities (pink dots) in a global analysis of microbial communities sampled in highly contrasted environments worldwide (dots). Principal Coordinates Analysis (PCoA) ordination is based on Bray–Curtis indices computed using the Qiita management platform. Note that two of the three herbarium samples located under the 0.0 value of the PCoA2 axis are two of the three communities from leaves (green triangles).

Integration of herbarium microbiomes into the 817 metagenomes collected for EMP in highly contrasted environments, including soil and plant organs, was performed to evaluate if conservation in herbaria altered the taxonomic diversity of their microbial communities or if they were inadvertently contaminated by communities from other environments. PCoA ordination of all metagenomes labelled by environment and project (EMP or herbarium) showed that soil and root herbarium samples grouped exclusively, albeit with a slight misalignment, with metagenomes from extant non‐saline soils and sediments. Herbarium‐associated metagenomes were clearly distinct from metagenomes of animals, aquatic environments (fresh or saline waters, saline sediments) and plant surfaces (aerial surfaces of vascular plants or surfaces of algae, Figure 2). Furthermore, none of the herbarium root or soil metagenomes appeared haphazardly distributed in the PCoA1‐2 projection space, as could be the case if long‐term conservation resulted in a random reshuffling of the original microbial assemblages. Two of the three herbarium leaf microbiomes and only one herbarium root microbiome did not tightly cluster with extant soil metagenomes.

### 3.3 Taxonomic Annotation and Alpha Diversity

**FIGURE 3:**
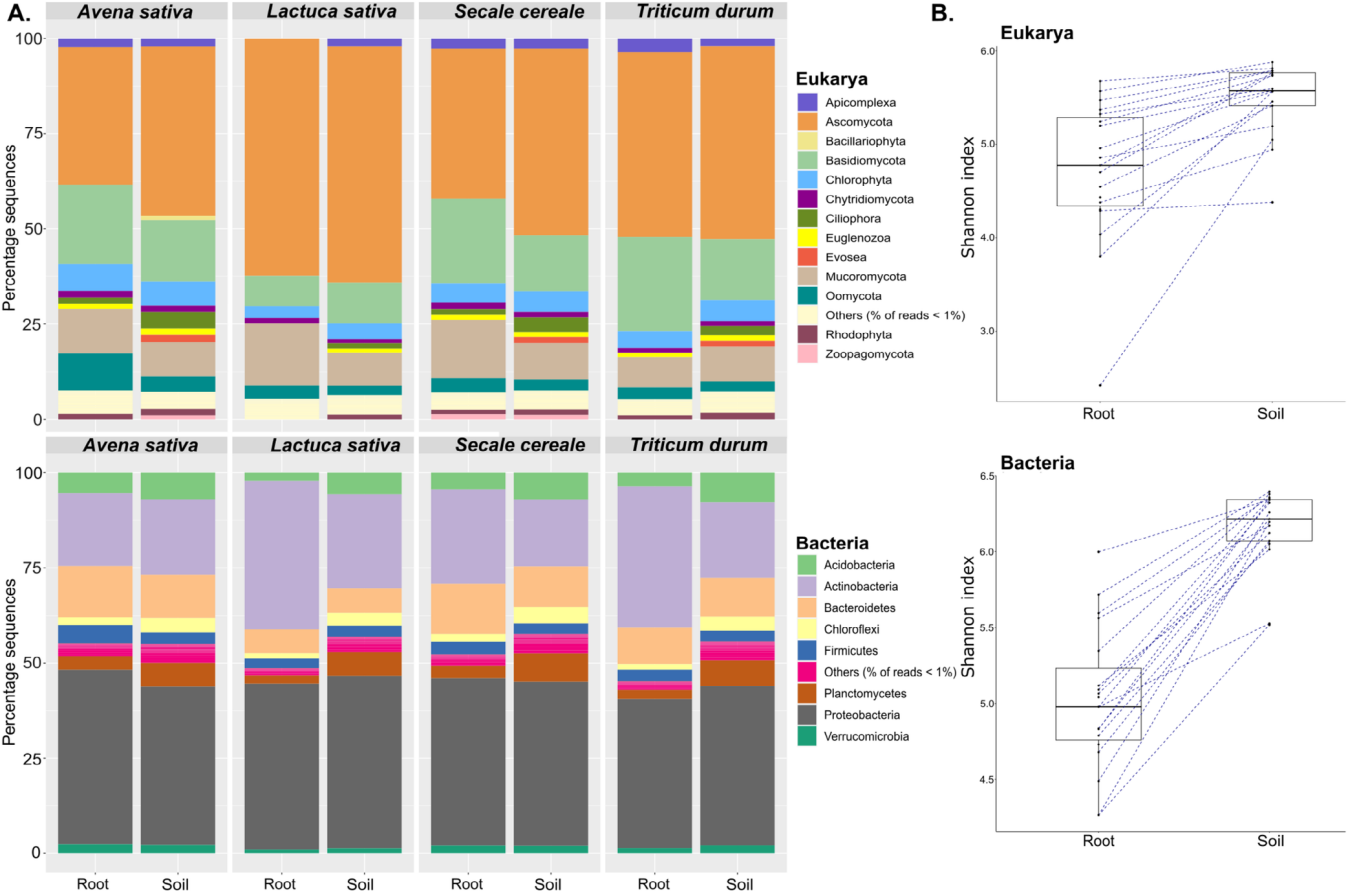
Taxonomic (alpha) diversity of herbarium‐associated soil and root microbial communities. (A) Taxonomic composition (phylum level) of eukaryotic (top) and bacterial (bottom) microbial communities associated with the roots or the soils of the four studied plant species. Each bar represents the mean value of the different replicates of samples collected in Saint Cloud garden in Paris (excluding samples collected from other collection sites). Phyla represented by < 1% of the total number of annotated sequences were pooled in a single category (Others). (B) Distribution of Shannon biodiversity indices (calculated at the genus level) of root and soil eukaryotic and bacterial communities. Dotted lines connect the values of the root and soil communities of each individual herbarium plant sample. *Lactuca* herbarium plant samples from which no soil samples could be collected were excluded from this analysis.

Using Kaiju, 85.2% of the microbiome sequences did not receive any taxonomic affiliation. At the phylum level, 95.82% of the annotated sequences were assigned to Bacteria, 2.74% to Eukarya, 1.36% to Archaea and 0.08% to viruses. A genus‐level assignation was obtained for 53.23% of the bacterial sequences and for 83.16% of the eukaryotic ones. The five most represented bacterial phyla were in the following order: Proteobacteria, Actinobacteria, Bacteroidetes, Planctomycetes, and Acidobacteria. Eukarya was mainly represented by fungi (essentially Ascomycota, Basidiomycota and Mucoromycota that encompassed symbiotic Glomeromycotina). More than 1% of the sequences were also affiliated to other unicellular eukaryotes (e.g., Oomycota, Ciliophora or Apicomplexa, Figure 3A). Since DNA was extracted from minute amounts of material, no Metazoa genome alignment was attempted.

Shannon alpha diversity indices calculated at the genus level were globally higher for soil compared to root samples (*p* < 0.01; Kruskal–Wallis). This global difference holds true for each individual herbarium plant sample (Figure 3B). At the genus level, in the case of bacteria from the collection site of Saint‐Cloud, a large proportion of the genera were identified in both soil and roots (53% and 71% for genera represented by at least 0.3% or 0.05% of the total number of reads, respectively). Genera identified only in soil outnumbered those identified only in roots (39 vs. 25 or 104 vs. 42 for genera represented by at least 0.3% or 0.05% of the total number of reads, respectively). A similar distribution pattern between roots and soil was also observed for eukaryotic taxa (Figure S6).

### 3.4 Beta Diversity Analyses

**FIGURE 4:**
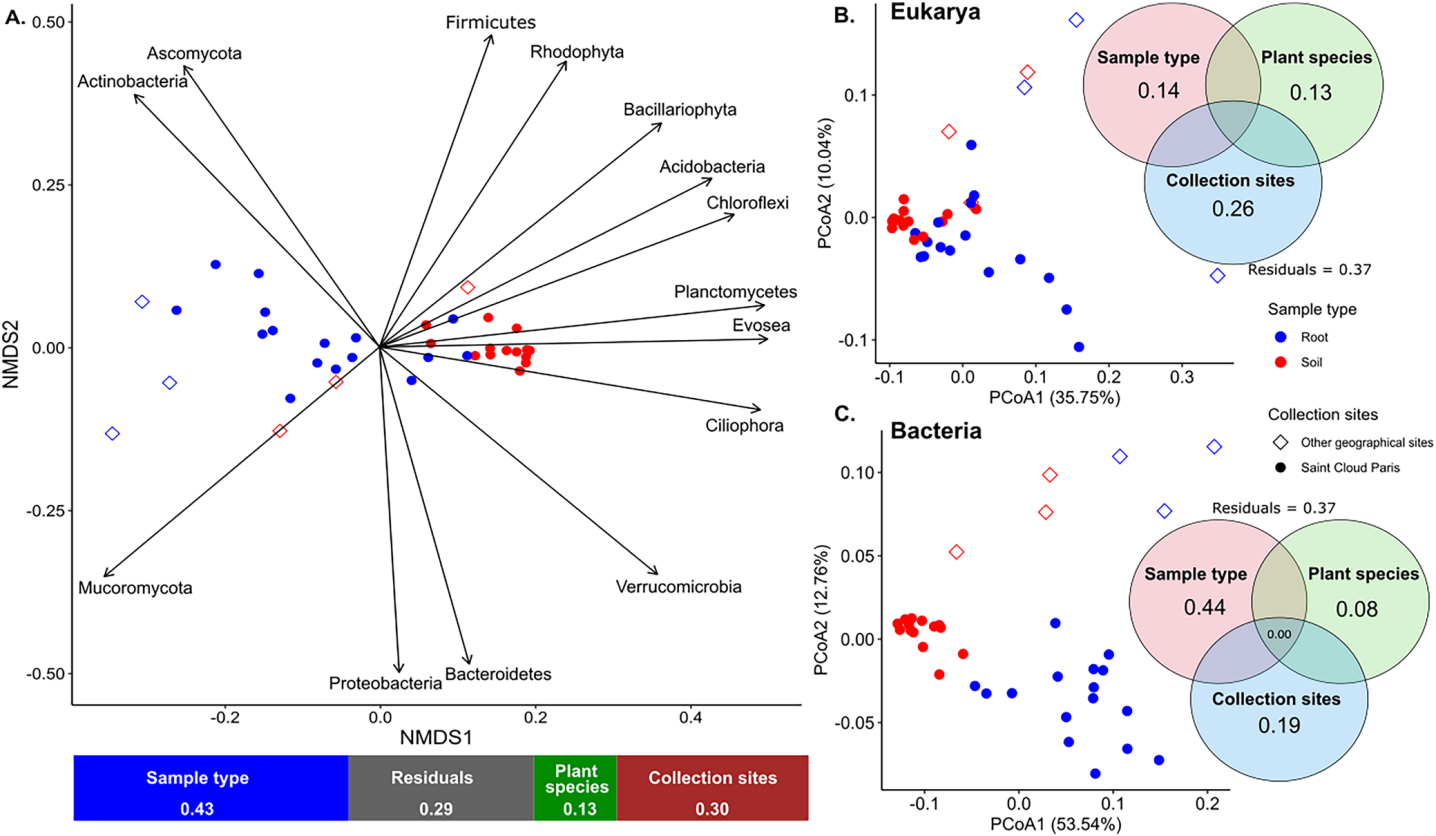
Sampling collection site and sample type (soil vs. root) affect herbarium‐associated communities at both the phylum and genus levels. (A) At the phylum level, non‐metric multidimensional scaling (NMDS, *k* = 3, stress < 0.05) ordination based on Bray–Curtis distances illustrates the distribution of microbial communities according to collection site and sample type (red: soil; blue: root; filled dots: garden of Saint‐Cloud, empty triangles: other sites). Explanatory variables (phyla represented by more than 1% of the reads assigned) that significantly (*p* < 0.05) contributed to the separation of the communities along the two axes are indicated. The lower bar gives the percentage of total variance explained by each variable. (B) and (C) Principal Coordinates Analysis ordinations based on Bray–Curtis distances illustrate the distribution of eukaryotic (B) and bacterial (C) communities according to collection site and sample type. Venn diagrams give the percentage of total variance explained by each of the variables and their interaction (only explained variance values > 0.01 are reported).

The factors that affected the microbial community structure were evaluated at both phylum and genus levels. At the phylum level, sample type (root vs. soil) had the strongest effect, over plant host species, collection site and interaction of these terms (*p* < 0.001, PERMANOVA). Clearly, microbial compositions from different sample types separate on the NMDS ordination (*k* = 3, stress < 0.05, Figure 4A, Table S5). The phyla Planctomycetes, Chloroflexi, Acidobacteria, Ciliophora, and, to a lesser extent, Verrucomicrobia, which were more abundant in soil, contributed most to the differences between soil and root communities (Figure 4A). Root communities tended to be slightly richer in fungal (Basidiomycota, Mucoromycota) and Actinobacteria sequences than soil ones (Figure 3A).

At the genus level, a similar sample type effect for both Bacteria and Eukarya on community composition was observed (*p* < 0.001, PERMANOVA, Table S5). PCoA ordination of the samples also illustrated the separation between the soil and root samples and, to some extent, the separation between root samples from different plant species (Figure S7). While the first axis of the PCoA ordination highlighted the differences between root and soil communities, the second axis separated soil and root communities of plants collected in Saint‐Cloud from soil and root communities of plants collected in the other three sites (Figure 4). Regarding plant species, for bacteria, both axes contributed to the separation of *Lactuca* and *Secale* root samples, while for Eukarya, both axes contributed to the separation of *Lactuca* samples from all other species' root samples (Figure S7). These analyses at the genus level were repeated using only the 12 most abundant bacterial genera for which the ancient origin of their DNA was evaluated. Reducing the dataset to these 12 taxa was sufficient to highlight statistically supported differences between soil and root communities, between samples collected in Saint‐Cloud and those collected in other sites, and between communities associated with *Lactuca* plants and those associated with cereal ones (Figure S8).

**FIGURE 5:**
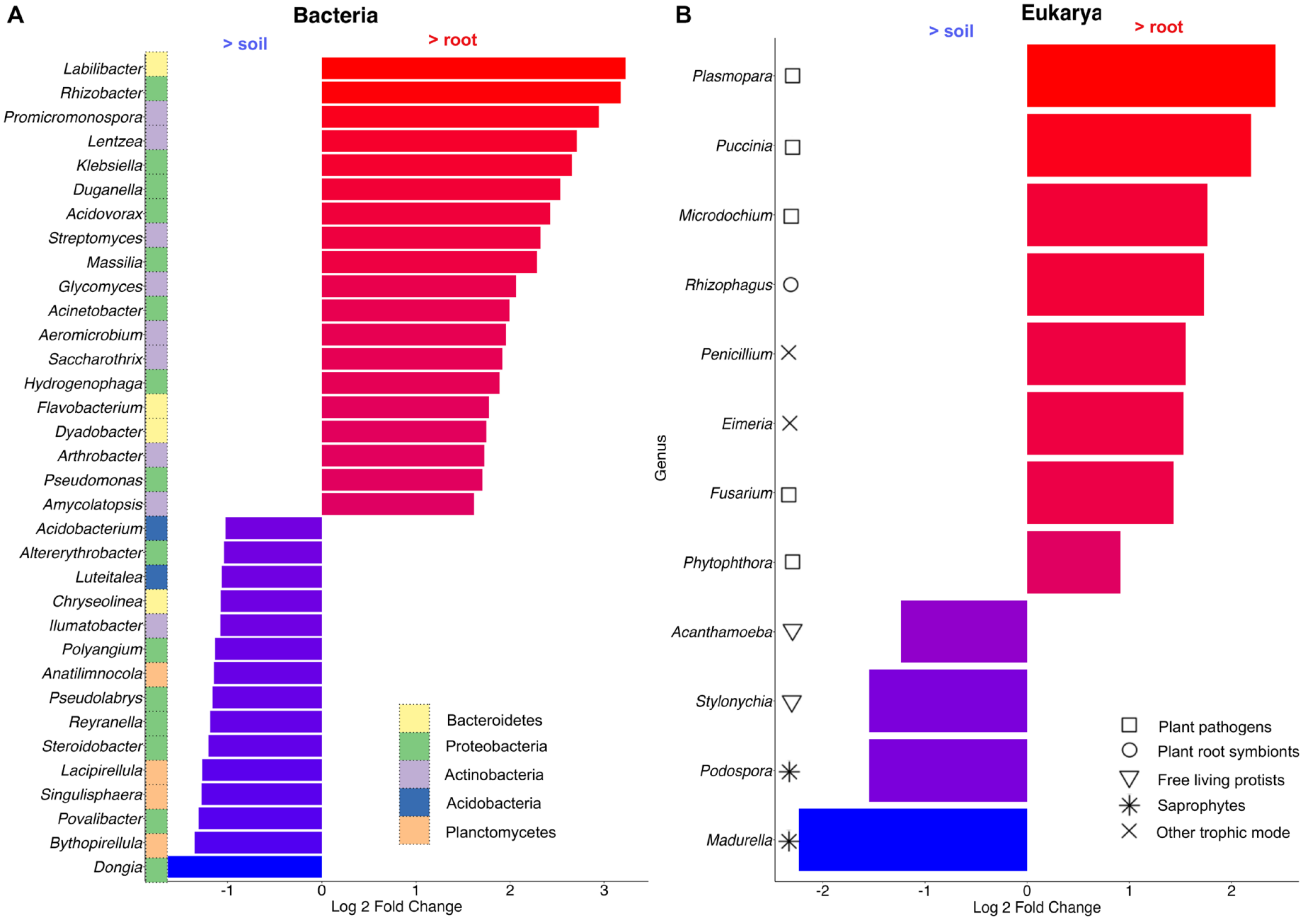
Bacterial and eukaryotic genera that showed differences in abundance between herbarium soil and root samples. Analysis using the DeSeq software allowed identifying taxa that displayed a difference in read abundance between the global root and soil sequence datasets above a threshold of 1.5/−1 for Bacteria and 0.9/−1 for Eukarya (Log_2_ scale). (A) Differentially abundant bacterial taxa (*p* < 0.001). Taxonomic annotation (phylum level) highlights differences between taxa displaying a higher abundance in soil (dominated by Planctomycetes) and those more abundant in roots (dominated by Proteobacteria and Actinomycetes). (B) Differentially abundant eukaryotic taxa (*p* < 0.05). Functional annotation highlights differences between taxa displaying a higher abundance in soil (unicellular protists and fungal saprotrophs) and those more abundant in roots (dominated by plant pathogens and symbionts). Since reads assigned to eukarya were far less abundant compared to those attributed to bacteria, the analysis was restricted to taxa with more than 50,000 and 5000 reads in the global dataset for bacteria and eukarya, respectively.

In the case of microbial genera, as recommended by Nearing et al. (2022), we implemented three alternative statistical approaches (DESeq2, ANCOMBC2 and ALDEx2) to identify taxa whose abundance differed between root and rhizospheric soil samples (Figure 5, Tables S7 and S8). The three approaches highlighted congruent taxonomic differences between soil and root‐enriched bacterial taxa. Thus, using DeSeq, while 8 of the 19 root‐enriched taxa belonged to Actinomycetes (42%), none belonged to Planctomycetes or Acidobacteria, which were identified in soil (6 of the 15 genera, i.e., 40%). Regarding Eukarya, although fewer genera were retained as being significantly enriched in either soil or roots, these two categories sharply differed with respect to their taxonomy, but also to their known patterns of interaction to plants. Taxa more abundant in roots were essentially obligate root symbionts (affiliated to the Glomeromycota) or known plant pathogens, or endophytes affiliated to the Oomycota (in the Peronosporales) or the Fungi (either Basidiomycota or Ascomycota). None of these categories related to plant–microbiome interactions were identified among the taxa found as more abundant in soil. These later taxa were mostly fungal genera known as soil saprotrophs or free‐living unicellular eukaryotes (belonging to, e.g., phyla Amoebozoa or Alveolata).

## 4 Discussion

Analysis of DNA extracted from herbarium soil and plant roots strongly supports our hypothesis that this material is suitable to evaluate the past diversity of soil microorganisms for the period under scrutiny (from at least the beginning of the 20th century). The extraction protocol, optimised for ancient sediment samples (Murchie et al. 2021), was effective in recovering DNA present in limited samples of both soil and roots from four collection sites and associated with four different plant species. Enzymatic manipulation of the DNA (with proteinase K during extraction and various enzymes and polymerases during library preparation) was not prevented by the historical treatments by trichloro(nitro)methane (chloropicrin) and Mercury(II)‐chloride used for preservation and disinfection (Staats et al. 2011; Herbarium of Lyon, personal communication).

DNA fragment length was on average below 50 bp, which is in the range of what has been reported for herbarium plants (Campos et al. 2023; Donegan et al. 2025; Särkinen et al. 2012; Staats et al. 2011) and is one of the typical characteristics of aDNA (Pääbo et al. 2004). However, the average length of the extracted DNA appears to be significantly affected upon the extraction sample type and slightly variable among the taxa considered. In most cases, including for the plant DNA, the fragments extracted from soil are longer than those extracted from plant tissues, either roots or leaves. This may result from a protective effect of the soil environment (salt content, presence of minerals adsorbing DNA, fast desiccation) over DNA fragmentation by depurination, a process known to be affected in vitro by different physico‐chemical parameters such as low pH, presence of water, metal ions or polycations (An et al. 2014, 2017). Regarding the ‘microbial taxon effect’ on DNA degradation, it seems to be linked to genome GC content; the higher the GC‐content, the higher the degradation (lower average fragment size). This phenomenon could result from a potentially greater depurination sensitivity for guanine compared to adenine residues (Kunkel 1984; Briggs et al. 2007). Alternatively, we cannot exclude that it could result from a technical bias that occurs during the preparation or the sequencing of the libraries. Illumina sequencing is known to be negatively affected by uneven GC content (Chen et al. 2013; Benjamini and Speed 2012), yet no significant effect of this parameter on the produced read length has ever been documented.

Beside the DNA fragmentation, we also highlighted the expected higher occurrence rates of cytosine deamination at both ends of the DNA fragments for the plant and the most abundant bacterial genera. Both these features are typical for ancient/historical DNA and suggest that extracted DNA is predominantly authentic, presumably not or marginally affected by recent contamination of the samples by extant microorganisms. This is an expected observation for herbarium samples that have usually been desiccated rapidly after collection and kept dry since then (Burbano and Gutaker 2023; Bieker and Martin 2018). Preservation of original microbiomes has been suggested in the case of human palaeofaeces, which may have taken, compared to herbarium plants, longer times to fully dry out in dry desert (Wibowo et al. 2021), cave (Hagan et al. 2020) or salt‐saturated (Maixner et al. 2021) environments. In addition, no positive amplification was obtained from any of the extraction and library control blanks, which excludes significant contamination of the samples during the laboratory experiments. We cannot exclude, however, that fluctuations in hygrometry during conservation may have allowed occasional secondary microbial colonisation of the soil and roots. Post‐mortem colonisation of herbarium plants was indeed hypothesised by Bieker et al. (2020) to explain the presence of several microbial taxa specifically identified on leaves of *Ambrosia* and *Arabidopsis* herbarium plants that were not observed in modern, fresh leaf samples.

Comparison of microbial DNA root and soil datasets to various microbiomes from highly contrasted environments showed that none of our samples behaved as outliers in the PCoA ordination, but almost all tended to cluster with modern soil samples and not with samples from other environments. Among the three samples that did not closely cluster with modern soil microbiomes, two corresponded to leaf samples. This is a further indication that secondary contamination of the samples was minimal and that preservation in herbaria did not grossly alter the relative proportions of the microorganisms that characterised the original communities. Similar observations were reported in the case, for example, of thousand years‐old human dental calculi whose microbial DNA contents were more similar to DNA extracted from modern human calculi than from other human‐associated microbiomes (Fellows Yates et al. 2021; Granehäll et al. 2021). These comparisons between different environments are crucial to detect possible microbial contamination during the preservation period and will need to be repeated on every new herbarium specimen before microbiome investigations.

We also observed that root microbial communities were always less diverse than their cognate soil ones and that taxa (genera) identified in roots were, for a majority of them, also present in soils. These observations are also in line with what is known of root microbial communities that are mostly recruited horizontally from the surrounding local pool of soil microorganisms (Edwards et al. 2015; Xun et al. 2021).

In terms of community structure, herbarium soil and root microbial communities also follow well‐described assembly rules. In the literature, the sampling site, with local specific climate and physico‐chemical features, is always the main factor affecting soil community composition (Peiffer et al. 2013). In the present study, our sampling design was highly unbalanced with regard to this factor; all samples from the ‘Saint‐Cloud’ site clearly grouped together in a PCoA ordination (genus level) and were clearly separated from the few samples collected in three other sites.

In plant microbiology, the second most important factor controlling microbial community composition is the sample type, in our case soil and root (Xiong et al. 2021). Once again, we observed a clear sample type effect on community composition at both phylum and genus levels. Visualisation of this effect was certainly facilitated by the analysis of herbarium plants that, for most of them, were grown in a single and restricted site (a garden in the town of Saint‐Cloud), thus minimising the confounding collection site effect. Furthermore, the very clear sample type effect was observed in spite of a potentially significant level of cross‐contamination between soil and root herbarium samples. Dried herbarium root samples cannot indeed be totally freed of adhering soil particles, as would be the case for fresh ones that are usually cleaned by sonication. At the phylum level, Acidobacteria and Chloroflexi were consistently more abundant in soils, as already observed in other studies that compared different plant species grown in different sites (Tkacz et al. 2020; Xiong et al. 2021). Soils were also globally enriched in Planctomycetes and Acidobacteria that predominated among genera found enriched in soil. At the genus level, functional roles assigned to eukaryotic taxa enriched in soil or roots were in line with their known associations to plants, with mostly pathogenic and mutualistic taxa among root‐enriched taxa that were completely absent among soil‐enriched ones that were free‐living unicellular eukaryotes and saprotrophic fungi. While symptoms of attacks by pathogens are difficult to record on dried roots covered by soil, it is worth emphasising that several of the root‐enriched taxa are biotrophic pathogenic Oomycetes or fungi, as well as mutualistic Glomeromycota, that need a living plant host for their growth and therefore cannot represent post‐harvest external secondary contaminants of the samples. In different herbarium root samples, typical cellular structures (arbuscules and vesicules) produced by Glomeromycota fungi were indeed observed under the microscope (Bianciotto et al. 2022 and unpublished observations).

Finally, a third, albeit less pronounced effect, is that of the plant species in the case of root communities. Although communities from all plant species could not be separated, it is worth mentioning that for both Bacteria and Eukarya, *Lactuca*‐associated communities differ from communities associated with the other plants that belong to the phylogenetically distant Poaceae family.

Altogether, these different observations support the hypothesis that herbarium material retains the original characteristics of the plant and soil‐associated microbiome as it was when the plants were collected. The approach can thus be extended to a larger number of samples to evaluate how cultivated soil microbiomes have been historically affected by the evolution of farming practices. Visual inspection of digitalised herbaria indicates that for several cultivated plant species, particularly cereals, numerous specimens have been preserved with associated rhizospheric soil. It is thus possible to analyse time series of different plants that cover a period of time that spans almost two centuries, the period during which most of the herbarium collections have been established.

## Author Contributions

R.M., V.B. and G.G. designed the research. R.D., G.G. and R.M. performed the research. G.G., L.B., F.L., M.A. and O.R. performed the data analysis. R.M., V.B. and G.G. wrote the paper, with the contribution of the other authors.

## Conflicts of Interest

The authors declare no conflicts of interest.

## Supporting information


Data S1

## Data Availability

Sequence datasets analysed during the current study are available in the EMBI‐ENA database under Bioproject PRJEB75398 (https://www.ebi.ac.uk/ena/browser/view/PRJEB75398). Datasets are also available on the Qiita platform under study ‘Herbaria microbiome project—ID 14959’ for comparison to other microbiome sequence data (https://qiita.ucsd.edu/).
