# Supplementary material for: Ancient Microbiomes as Mirrored by DNA Extracted From Century‐Old Herbarium Plants and Associated Soil
Source: Mol Ecol Resour. 2025 May 24;25(7):e14122. doi: 10.1111/1755-0998.14122 (PMC12415840; doi:10.1111/1755-0998.14122)
Supplement: Supplementary file 1 — Data S1 [file MEN-25-e14122-s001.zip › men14122-sup-0001-Figures.docx]

**Ancient microbiomes as mirrored by DNA extracted from century-old herbarium plants and associated soil**

Gianluca Grasso^1,2,3,*,†^, Régis Debruyne^4,*^, Martino Adamo^1^, Olivier Rué ^5^, Franck Lejzerowicz^6^, Lucie Bittner^2,7,#^, Valeria Bianciotto^3,#^, Roland Marmeisse^2,3,#^

**Supplementary Figure and Table legends**

- Figure S1. Summary of Sequencing Data Analysis
- Figure S2. Length distribution profiles of plant sequences.
- Figure S3. Length distribution of 12 more abundant taxa sequences.
- Figure S4. Deamination profiles of plant sequences.
- Figure S5. Deamination profiles of 12 more abundant taxa sequences.
- Figure S6. Genera shared in root and soil herbarium microbiomes.
- Figure S7. Effect of plant species on bacterial and eukaryotic root microbiomes.
- Figure S8. Effect of plant species, matrix and geographic origins on 12 most abundant taxa.
- Table S1. Studied herbarium material.
- Table S2. References nuclear, mitochondrial, and plastid plant genomes.
- Table S3. Reference genomes of the 12 bacterial genera used to run MapDamage.
- Table S4. Detailed description of the processing of the sequence datasets.
- Table S5. PerMANOVA analysis of Eukarya and Bacteria beta diversity (phylum and genus levels).
- Table S6. PerMANOVA analysis of Eukarya and Bacteria beta diversity of root microbiomes.
- Table S7. Eukaryotic differential abundance analysis with ANCOM-BC2 and ALDEx2.
- Table S8. Bacterial differential abundance analysis with ANCOM-BC2 and ALDEx2.

**Figure S1. Summary of Sequencing Data Analysis A.** Total number of sequences obtained after demultiplexing, quality controls, merging and eliminations of reads shorter than 25 bp of the raw data. In millions of sequencing reads, the filtered data had an average of 21.8, a standard deviation of 7.7, a minimum of 8.5 for sample Sc707 and Sc-689 rhizospheric soil and a maximum of 40.3 for sample Td-279 root. **B.** Average length (bp) value of the merged reads of the samples. Average values of the different samples are 40.72 for leaf, 48.02 for rhizospheric soil and 42.14 for root samples.


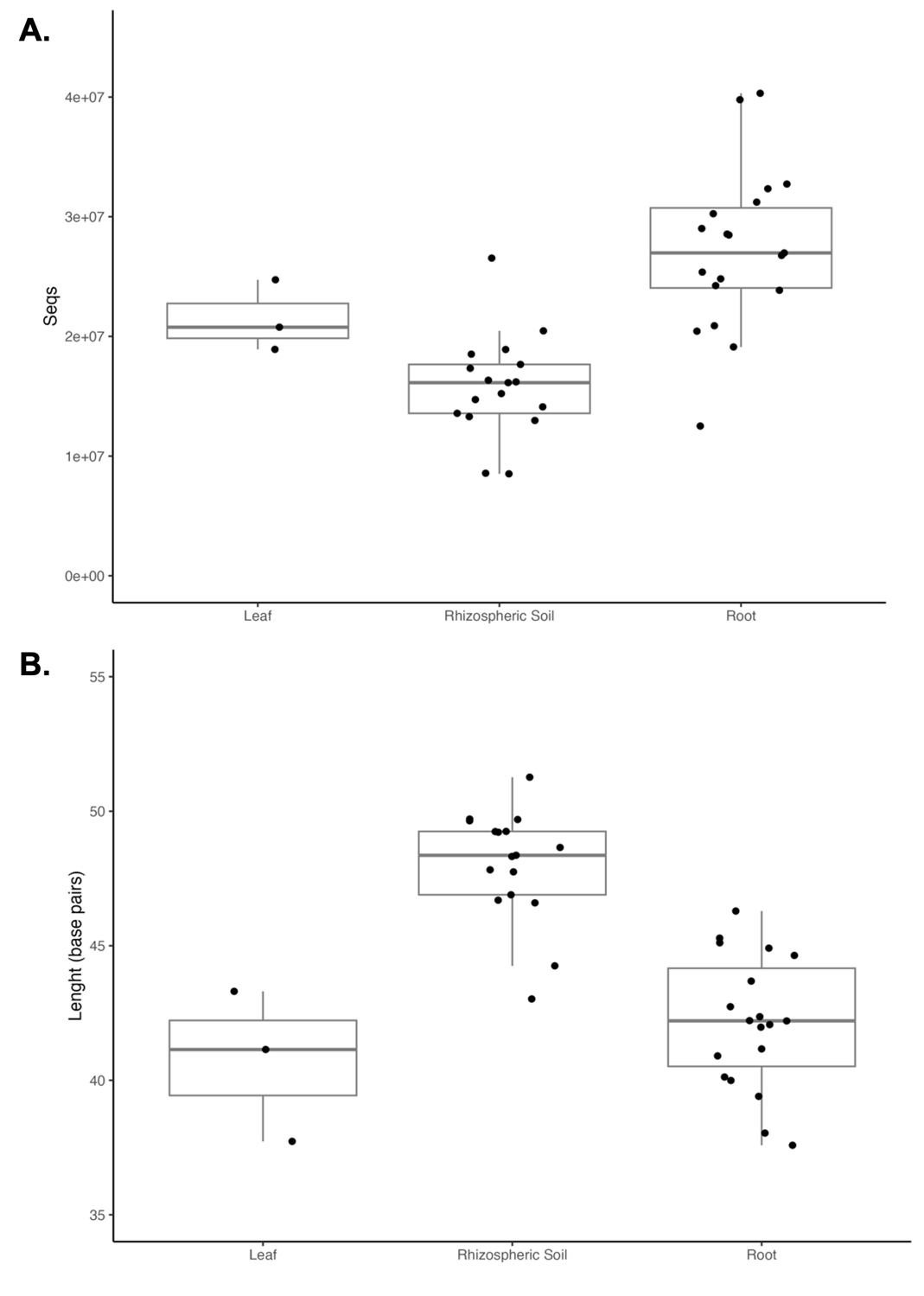


**Figure S2. Length distribution profiles of plant sequences.** Length distribution of DNA fragments (merged reads) extracted from roots (blue curves) and associated soil (in red) affiliated to the plants species (A. sativa, L. sativa, S. cereale and T. durum). Deamination profiles are illustrated for the first 25 nucleotides at the 5’ end (G to A transitions) and 3’ one (C to T transitions).

**Figure S3. Length distribution of 12 more abundant taxa sequences**. Length distribution of DNA fragments (merged reads) extracted from roots (blue curves) and associated soil (in red) affiliated to the more abundant genera (Streptomyces, Nocardioides, Pseudomonas, Flavobacterium, Bradyrhizobium, Lentzea, Microbacterium, Sphingomonas, Massilia, Devosia, Variovorax and Rhizobium). Deamination profiles are illustrated for the first 25 nucleotides at the 5’ end (G to A transitions) and 3’ one (C to T transitions).

**Figure S4. Deamination profiles of plant sequences.** Deamination profiles of DNA fragments (merged reads) extracted from roots (blue curves) and associated soil (in red) affiliated to the plants species (A. sativa, L. sativa, S. cereale and T. durum). Deamination profiles are illustrated for the first 25 nucleotides at the 5’ end (G to A transitions) and 3’ one (C to T transitions).


**Figure S5. Deamination profiles of 12 more abundant taxa sequences.** Deamination profiles of DNA fragments (merged reads) extracted from roots (blue curves) and associated soil (in red) affiliated to the more abundant genera (*Streptomyces, Nocardioides, Pseudomonas, Flavobacterium, Bradyrhizobium, Lentzea, Microbacterium, Sphingomonas, Massilia, Devosia, Variovorax* and *Rhizobium*). Deamination profiles are illustrated for the first 25 nucleotides at the 5’ end (G to A transitions) and 3’ one (C to T transitions).

**Figure S6. Genera shared in root and soil herbarium microbiomes from Saint Cloud garden.**  At the genus level, in the case of bacteria (**A**) and the samples from Saint-Cloud, a large proportion of the genera were identified in both soil and roots (53 % and 71 % for genera represented by at least 0.05 % (left) or 0.3 % (right) of the total number of reads, respectively). Genera identified only in soil outnumbered those identified only in roots (39 *versus* 25 or 104 *versus* 42 for genera represented by at least 0.3 % or 0.05 % of the total number of reads, respectively). A similar pattern of distribution of taxa between roots and soil was also observed for Eukarya for which the relaxed and stringent filtering thresholds were set to 0.1 (right) and 0.5 % (left) of the total number of reads"(**B**).


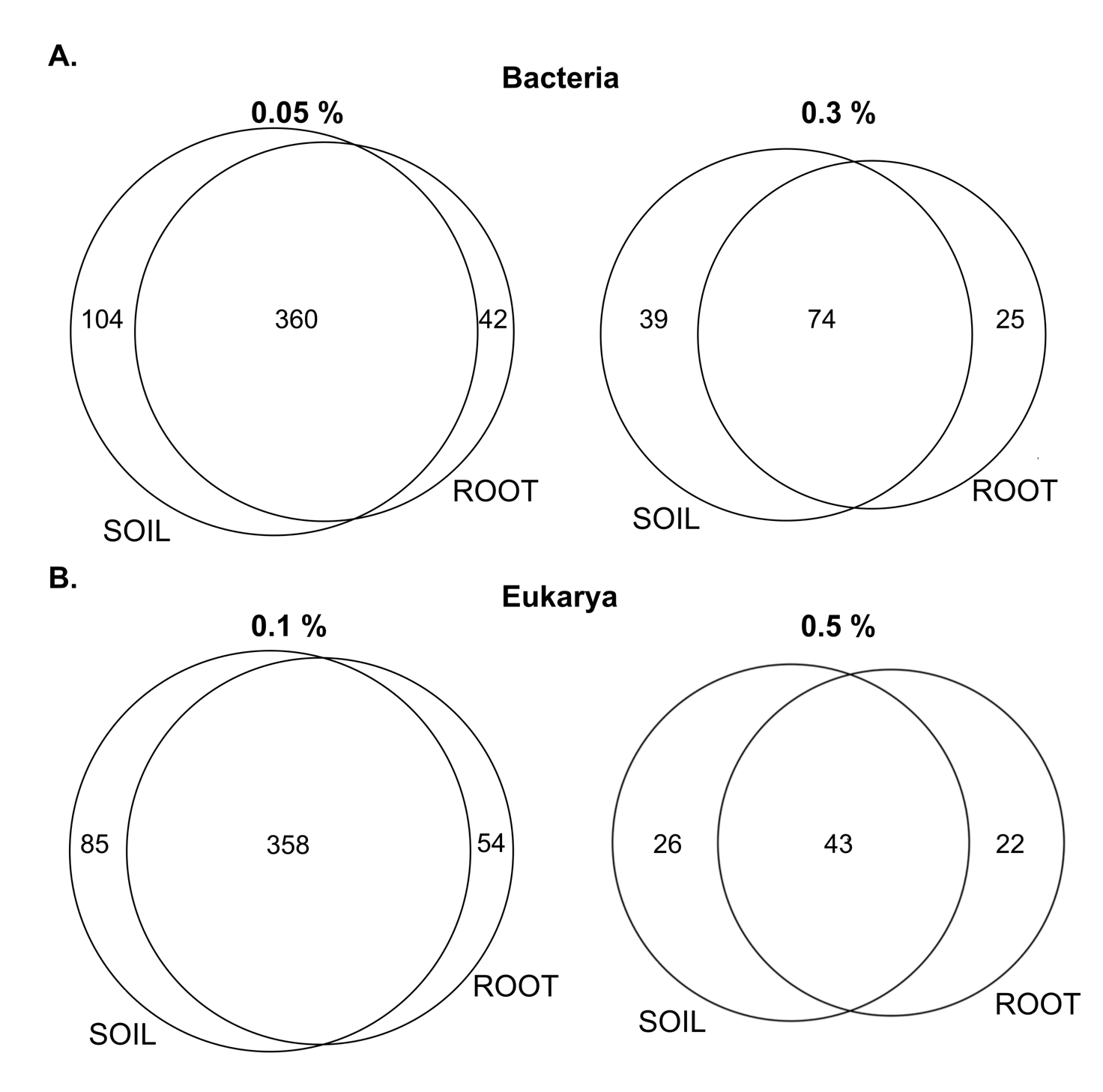


**Figure S7. Effect of plant species on bacterial and eukaryotic root microbiomes.** Principal Coordinates Analysis ordinations based on Bray-Curtis indices illustrate the distribution of eukaryotic (A) and bacterial (B) root communities according to the plant species (P< 0.05, PerMANOVA, **Table S6**).


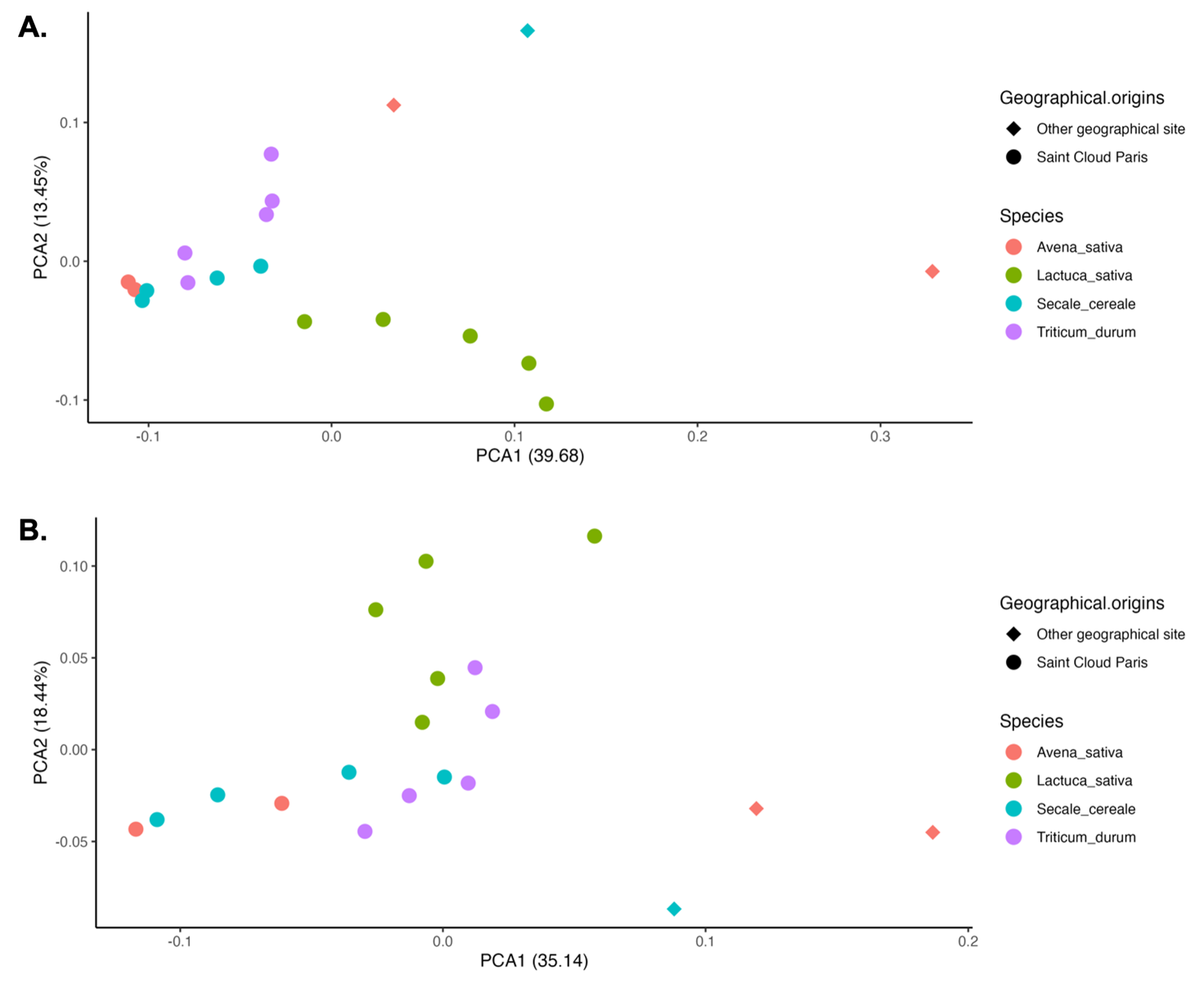


**Figure S8. Effect of plant species (A) and sample type (root vs. soil) (B) on bacterial microbiomes.** Principal Coordinates Analysis ordinations based on Bray-Curtis indices computed using a dataset limited to the 12 most abundant bacterial genera (as listed in Fig. S5) for which DNA degradation patterns were ascertained (Fig. S5). The table below presents all the parameters obtained from PerMANOVA analyses on the 12 most abundant Bacteria beta diversity matrix. Microbial communities were significantly diverse among sample types (root and rhizospheric soil), geographic origins and plant species.

**A.**

**B.**

**Table S1. Studied herbarium material.** All samples came from the herbarium of the University Lyon 1 (LY), France. “species names”, “poisoned”, “variety”, “collection date” and “collection site” refer to information written on the herbarium sheets; “ni”, not indicated. Plants from Saint-Cloud were grown in a common garden that no longer exists due to urbanization of the area. “Specimen accession No.” refers to the Recolnat accession Nos. that allow visualizing the corresponding material (<https://explore.recolnat.org/search/botanique/type=index>).

**Table S2. References nuclear, mitochondrial, and plastid plant genomes** . All plant reference genomes were downloaded from the NCBI database. For species whose complete mitochondrial or chloroplast genomes were not available, genomes of phylogenetically-close plant species were selected.

**Table S3. Reference genomes of the 12 bacterial genera used to run MapDamage.** MapDamage was run using the metagenomics sequences that did not map on plant genomes using as reference database all of the reference genomes (RefSeq present in the NCBI database of April 2023) affiliated to the 12 most abundant bacterial genera identified following taxonomic affiliation of the sequences using Kaiju.

Large table attached singularly *(file: Table_S3_bacterial_genomes*)

**Table S4. Detailed description of the processing of the sequence datasets.** Table includes information such as sample type, the number of merged reads, indexing PCR cycles, mapped sequences on plant genomes (Table S2), mapped reads on the h28 human reference genome, total numbers, percentages of mapped reads, and unmapped reads.

**Table S5. PerMANOVA analysis of Eukarya and Bacteria beta diversity (phylum and genus levels).** The table presents all the parameters obtained from PerMANOVA analyses on Eukarya and Bacteria beta diversity matrices. Microbial communities, both at genus and phylum level, were significantly diverse among sample types (root and rhizospheric soil), geographic origins and plant species. In the tables are reported degree of freedom (df), sum of squares, coefficients of determination (R2), F statistic value (F), P-value (Pr(>F)).

**Table S6. PerMANOVA analysis of Eukarya and Bacteria beta diversity of root microbiomes.**

The table presents all the parameters obtained from PerMANOVA analyses on Eukarya and Bacteria beta diversity matrices limited to root microbiome samples. Root microbiome communities, at genus level, were significantly different among sample types (root and rhizospheric soil) geographic, origins and plant species. In the tables are reported degree of freedom (df), sum of squares, coefficients of determination (R2), F statistic value (F), P-value (Pr(>F)).

**Table S7.** **Eukaryotic differential abundance analysis at the genus level using ANCOM-BC2 and ALDEx2.** Threshold used to select differentially abundant microbial genera in either the soil or the root compartments: ANCOM-BC2, q-value < 0.05; ALDEx2 we.eBH value < 0.05.

| Eukarya - ANCOM-BC2 | | | | | |  |
| --- | --- | --- | --- | --- | --- | --- |
| **Fold change** | **p_val** | **q_value** | **Taxon** | **Association** | **Trophic types** |  |
| 0.5 | 3.67E-05 | 0.03666425 | Stylonychia | High SOIL | Free living protists | * |
| 0.4 | 9.93E-06 | 1.01E-02 | Podospora | High SOIL | Saprophyte | * |
| 0.3 | 7.47E-12 | 8.16E-09 | Acanthamoeba | High SOIL | Free living protists | * |
| 0.3 | 6.83E-07 | 7.16E-04 | Ascobolus | High SOIL | Saprophyte |  |
| 0.2 | 2.33E-11 | 2.54E-08 | Planoprotostelium | High SOIL | Free living protists |  |
| 0.2 | 4.72E-05 | 0.04700212 | Naegleria | High SOIL | Free living protists |  |
| 0.2 | 4.47E-07 | 4.70E-04 | Halteria | High SOIL | Free living protists |  |
| 0.2 | 1.64E-05 | 0.01656604 | Thermothielavioides | High SOIL | Saprophyte |  |
| -0.1 | 5.35E-07 | 5.62E-04 | Melampsora | High ROOT | Plant pathogen |  |
| -0.3 | 1.65E-05 | 0.01667485 | Plasmopara | High ROOT | Plant pathogen | * |
| Eukarya - ALDEx2 | | | | | |  |
| **Effect** | **we.eBH** | **wi.eBH** | **Taxon** | **Association** | **Trophic types** |  |
| 1.1 | 0.009777994 | 0.01056592 | Ascobolus | High SOIL | Saprophyte |  |
| 1.1 | 0.012700098 | 0.01197416 | Planoprotostelium | High SOIL | Free living protists |  |
| 1.1 | 0.009407652 | 0.01193552 | Acanthamoeba | High SOIL | Free living protists | * |
| 1.1 | 0.01179484 | 0.013306 | Halteria | High SOIL | Free living protists |  |
| 1.0 | 0.01638579 | 0.01657237 | Letharia | High SOIL | Lichenicolous fungus |  |
| 1.0 | 0.011452383 | 0.01315144 | Stentor | High SOIL | Free living protists |  |
| 1.0 | 0.016571241 | 0.01282141 | Naegleria | High SOIL | Free living protists |  |
| -1.0 | 0.029154414 | 0.0180638 | Melampsora | High ROOT | Plant pathogen |  |
| -1.0 | 0.013880209 | 0.01279492 | Plasmopara | High ROOT | Plant pathogen | * |

**Table S8. Bacterial differential abundance analysis at the genus level using ANCOM-BC2 and ALDEx2.** Threshold used to select differentially abundant microbial genera in either the soil or the root compartments: ANCOM-BC2 q-value < 0.05; ALDEx2 we.eBH value < 0.05.

| Bacteria - ANCOM-BC2 | | | | | |  |
| --- | --- | --- | --- | --- | --- | --- |
| **lcf** | **p_val** | **q_value** | **Taxon** | **Association** | **Phylum** |  |
| 0.427 | 4.46E-07 | 0.00107187 | Steroidobacter | High SOIL | [Proteobacteria](https://en.wikipedia.org/wiki/Pseudomonadota) | * |
| 0.370 | 2.48E-12 | 6.83E-09 | Chryseolinea | High SOIL | Bacteroidetes | * |
| 0.353 | 1.08E-15 | 3.12E-12 | Ilumatobacter | High SOIL | Actinobacteria | * |
| 0.328 | 1.59E-25 | 5.05E-22 | Lacipirellula | High SOIL | [Planctomycetes](https://en.wikipedia.org/wiki/Planctomycetota) | * |
| 0.257 | 9.18E-07 | 0.00217817 | Luteitalea | High SOIL | [Acidobacteriota](https://es.wikipedia.org/wiki/Acidobacteriota) | * |
| 0.243 | 1.51E-10 | 3.98E-07 | Altererythrobacter | High SOIL | [Proteobacteria](https://en.wikipedia.org/wiki/Pseudomonadota) | * |
| 0.226 | 1.68E-09 | 4.31E-06 | Bythopirellula | High SOIL | Planctomycetes | * |
| 0.217 | 3.01E-15 | 8.69E-12 | Povalibacter | High SOIL | [Bacteroidota](https://en.wikipedia.org/wiki/Bacteroidota) | * |
| 0.216 | 1.38E-16 | 4.05E-13 | Reyranella | High SOIL | [Proteobacteria](https://en.wikipedia.org/wiki/Pseudomonadota) | * |
| 0.214 | 1.73E-20 | 5.32E-17 | Mesorhizobium | High SOIL | [Proteobacteria](https://en.wikipedia.org/wiki/Pseudomonadota) |  |
| -0.326 | 2.96E-06 | 0.00688127 | Devosia | High ROOT | [Proteobacteria](https://en.wikipedia.org/wiki/Pseudomonadota) |  |
| -0.339 | 1.38E-07 | 3.40E-04 | Acidovorax | High ROOT | [Proteobacteria](https://it.wikipedia.org/wiki/Proteobacteria) | * |
| -0.460 | 1.79E-05 | 0.04038731 | Duganella | High ROOT | [Proteobacteria](https://it.wikipedia.org/wiki/Proteobacteria) | * |
| -0.575 | 1.70E-05 | 0.0384703 | Promicromonospora | High ROOT | [Actinobacteria](https://it.wikipedia.org/wiki/Actinobacteria) | * |
| -0.623 | 4.28E-07 | 0.00103036 | Massilia | High ROOT | [Proteobacteria](https://it.wikipedia.org/wiki/Proteobacteria) | * |
| -0.903 | 3.66E-11 | 9.82E-08 | Streptomyces | High ROOT | [Actinobacteria](https://it.wikipedia.org/wiki/Actinobacteria) | * |
| Bacteria - ALDEx2 | | | | | |  |
| **Effect** | **we.eBH** | **wi.eBH** | **Taxon** | **Association** | **Phylum** |  |
| 1.703695413 | 2.12E-04 | 3.75E-04 | Parafilimonas | High SOIL | Bacteroidetes |  |
| 1.455019959 | 5.16E-04 | 3.26E-04 | Caulifigura | High SOIL | Planctomycetes |  |
| 1.332164313 | 6.79E-04 | 4.16E-04 | Gemmata | High SOIL | Planctomycetes |  |
| 1.321502343 | 6.15E-04 | 3.99E-04 | Povalibacter | High SOIL | Proteobacteria | * |
| 1.29579179 | 6.39E-04 | 3.87E-04 | Urbifossiella | High SOIL | Planctomycetes |  |
| 1.278044928 | 6.41E-04 | 3.93E-04 | Planctomyces | High SOIL | Planctomycetes | * |
| 1.266933006 | 7.20E-04 | 4.51E-04 | Frigoriglobus | High SOIL | Planctomycetes |  |
| 1.266823002 | 6.37E-04 | 3.61E-04 | Lacipirellula | High SOIL | Planctomycetes | * |
| 1.266618506 | 6.35E-04 | 3.85E-04 | Cyclobacterium | High SOIL | Bacteroidetes |  |
| -0.955775996 | 0.00186072 | 0.00379013 | Duganella | High ROOT | Proteobacteria | * |
| -0.956448529 | 0.00182307 | 0.0017182 | Promicromonospora | High ROOT | Actinobacteria | * |
| -1.064243635 | 9.05E-04 | 8.10E-04 | Streptomyces | High ROOT | Actinobacteria | * |
| -1.179085024 | 6.95E-04 | 4.48E-04 | Acidovorax | High ROOT | Proteobacteria | * |
